# Connectome of the Suprachiasmatic Nucleus: New Evidence of the Core-Shell Relationship

**DOI:** 10.1523/ENEURO.0205-18.2018

**Published:** 2018-10-02

**Authors:** Shruti Varadarajan, Mary Tajiri, Rashi Jain, Rebecca Holt, Qanetha Ahmed, Joseph LeSauter, Rae Silver

**Affiliations:** 1 Department of Psychology, Barnard College, New York, NY 10027,; 2 Departments of Psychology, Columbia University, New York, NY, 10027 and of Pathology and Cell Biology, Columbia University, New York, NY 10032

**Keywords:** circadian rhythm, connectome, gastrin-releasing peptide, suprachiasmatic nucleus, vasoactive intestinal polypeptide, vasopressin

## Abstract

A brain clock, constituted of ∼20,000 peptidergically heterogeneous neurons, is located in the hypothalamic suprachiasmatic nucleus (SCN). While many peptidergic cell types have been identified, little is known about the connections among these neurons in mice. We first sought to identify contacts among major peptidergic cell types in the SCN using triple-label fluorescent immunocytochemistry (ICC). To this end, contacts among vasoactive intestinal polypeptide (VIP), gastrin-releasing peptide (GRP), and calretinin (CALR) cells of the core, and arginine vasopressin (AVP) and met-enkephalin (ENK) cells of the shell were analyzed. Some core-to-shell and shell-to-core communications are specialized. We found that in wild-type (WT) mice, AVP fibers make extremely sparse contacts onto VIP neurons but contacts in the reverse direction are numerous. In contrast, AVP fibers make more contacts onto GRP neurons than conversely. For the other cell types tested, largely reciprocal connections are made. These results point to peptidergic cell type-specific communications between core and shell SCN neurons. To further understand the impact of VIP-to-AVP communication, we next explored the SCN in VIP-deficient mice (VIP-KO). In these animals, AVP expression is markedly reduced in the SCN, but it is not altered in the paraventricular nucleus (PVN) and supraoptic nucleus (SON). Surprisingly, in VIP-KO mice, the number of AVP appositions onto other peptidergic cell types is not different from controls. Colchicine administration, which blocks AVP transport, restored the numbers of AVP neurons in VIP-KO to that of WT littermates. The results indicate that VIP has an important role in modulating AVP expression levels in the SCN in this mouse.

## Visual Abstract

**Figure:**
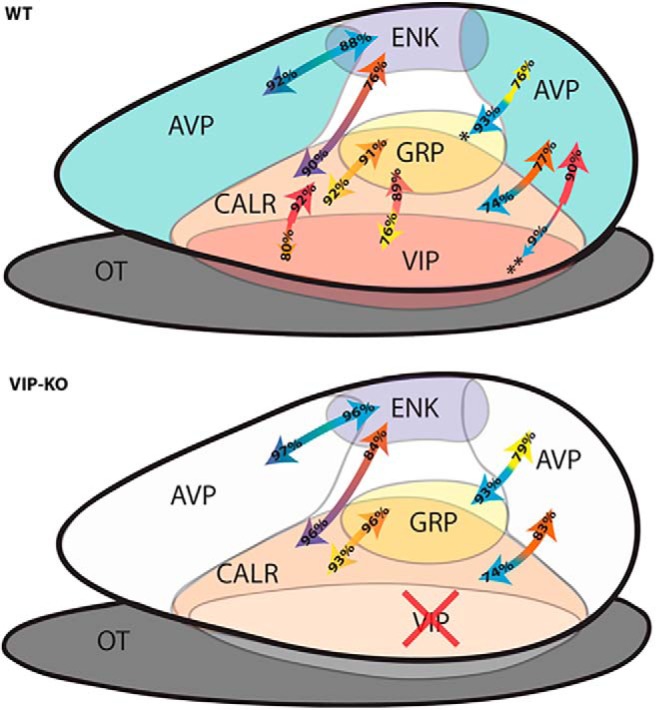

## Significance Statement

The suprachiasmatic nucleus (SCN) brain clock bears ∼20,000 neurons of many peptidergic types. Numerous studies have focused broadly on the core [vasoactive intestinal polypeptide (VIP)-rich]-to-shell [arginine vasopressin (AVP)-rich] areas, leaving aside questions of interconnections among specific peptidergic neuron types. Here, we describe contacts between AVP, gastrin-releasing peptide (GRP), VIP, met-enkephalin (ENK), and calretinin (CALR) SCN peptidergic phenotypes in wild-type (WT) mice and examine changes to these connections consequent to loss of the key core peptide, VIP. Thus, in the absence of VIP, expression of the shell peptide AVP was reduced. The work delineates interrelationships among specific SCN neurons and highlights a complexity in interpreting the consequences of single gene knock-outs.

## Introduction

It is well established that the suprachiasmatic nucleus (SCN) is a master brain clock, made up of many peptidergic neuronal types, and that the various SCN subregions are characterized by clusters of similar peptidergic cells (Antle and Silver, 2005; Yan et al., 2007). Among the best studied cell types of these SCN subregions are neurons synthetizing arginine vasopressin (AVP) in the shell or dorsal region, and those expressing vasoactive intestinal polypeptide (VIP) in the core or ventral region (Abrahamson and Moore, 2001; Moore et al., 2002; Antle and Silver, 2005). Also found in the shell are met-enkephalin (ENK)-expressing neurons and in the core are calretinin (CALR) and gastrin-releasing peptide (GRP)-producing neurons (Silver et al., 1999; Abrahamson and Moore, 2001). Possibly due to the small size of SCN neurons and its fine afferent and efferent fibers, there is relatively little known about the connectivity among these diverse neuronal cell types and how these might be altered in arrhythmic mutant animals.

### Importance of networks

As for other brain regions, it is generally accepted that the connectivity and characteristics of its neurons play an important role in the SCN functional specialization. Although individual SCN neurons are autonomous oscillators, they must function within a network to generate circadian rhythmicity within the nucleus (Welsh et al., 2010). There is limited information available on connectivity among SCN neurons. Early studies in rat using Golgi and Nissl staining in light and electron microscopic work demonstrated that the axons and dendrites of the greatest majority of its neurons terminate within the SCN (Güldner, 1976, 1984; van den Pol, 1980; Güldner, 1983; van den Pol and Tsujimoto, 1985; Klein et al., 1991). Güldner (1976) estimated that each SCN neuron made 300–1000 synaptic contacts. Subsequent work confirmed this assessment and estimated that within the SCN, individual neurons make as many as 1000 synapses (Moore and Bernstein, 1989). There is evidence of substantial communication within neurons of a specific cell type in that boutons immunoreactive for AVP synapse onto AVP containing dendrites and similarly, boutons containing GRP synapse onto GRP cells (van den Pol and Gorcs, 1986). Taken together, the weight of evidence indicates that network organization and communication among neurons, is key to the functioning of the brain clock, although little work has been done on the connections among SCN cell types.

### Connections of SCN cell types

There have been a few studies of contacts among SCN peptidergic cell types in the rat and hamster but none in mice, to our knowledge. In rats, using double-label immunochemistry, reciprocal appositions have been described between neurons bearing the following peptides: AVP and VIP (Ibata et al., 1993; Jacomy et al., 1999); AVP, VIP, and GRP (Romijn et al., 1997).

In hamsters, injection of tracers into the SCN indicates that the dorsal and medial regions project densely to most of the nucleus, but not to the core region, which had been identified by calbindin (CalB) neurons (Kriegsfeld et al., 2004). Interconnections among CalB and other peptidergic cells of the SCN have been examined using epi- and confocal microscopy and intra-SCN tract tracing (LeSauter et al., 2002, 2009).

Dynamic aspects of network organization have also been described. There is a rhythm in CalB expression in fibers, with many appositions seen between GRP and AVP at zeitgeber time (ZT)14 but exceedingly few at ZT4 (LeSauter et al., 2009). A similar finding has been reported in rats where more CALR fibers are typically seen at ZT14 than at ZT2 (Moore, 2016). To explore the intra-SCN network in which GRP neurons participate in more detail, individual GRP neurons bearing green fluorescent protein (GFP) were filled with biocytin tracer (Drouyer et al., 2010). These neurons form a dense network of local circuits within the core, revealed by appositions on other GFP-containing cells and by the presence of dye-coupled cells. Dendrites and axons of GRP cells make appositions on AVP neurons, whereas adjacent biocytin tracer-filled, non-GRP cells have a less extensive fiber network, largely confined to the region of GRP-bearing cells. This work in rats and hamsters points to the highly specialized connectome of the SCN and to its temporal dynamics.

### Importance of core and shell communication

The anatomic division of SCN into core and shell regions, based on the location of AVP and VIP-containing neurons aligns well with its functional organization. VPAC2, the receptor for VIP, is abundant in the SCN (Cagampang et al., 1998) and is essential in maintaining intercellular and behavioral rhythmicity in the SCN. Mice bearing a null mutation of the VPAC2 receptor cannot sustain normal circadian activity rhythms (Harmar et al., 2002; Piggins and Cutler, 2003; Maywood et al., 2006). In accord with the implications of this finding, mice lacking VIP in the SCN have abnormal circadian activity, impaired cellular rhythmicity, and reduced synchrony among neurons (Aton et al., 2005; Brown et al., 2007). In arrhythmic mice lacking essential components of the circadian transcription-translation feedback loop, the introduction of VIP signaling is sufficient to coordinate gene expression and maintain rhythmicity (Maywood et al., 2011). Such findings support the hypothesis that VIP originating in the core SCN, acting through its receptor, is crucial for maintaining rhythmicity.

The present study was designed to examine the connectome of the mouse SCN, as has previously been done in rats and hamsters. Given the importance of VIP and AVP in regulating circadian rhythmicity, a second goal was to assess changes in the network organization consequent to the loss of VIP. Thus, we examined sagittal and coronal sections of the SCN and used triple-label immunocytochemistry (ICC) and confocal microscopy, to examine wild-type (WT) and VIP-KO mice. We stained for neurons of the core, namely GRP, VIP, and CALR as well as those of the shell, specifically AVP and ENK. The contacts between various peptidergic types were quantified by determining the number of appositions between fibers of one peptidergic type onto the cell body of another. The results present a first analysis of the mouse SCN connectome network that leads to the generation of the circadian rhythm. The results also describe a vital role for VIP in determining AVP expression levels.

## Materials and Methods

### Animals and housing

Two genotypes of male animals were used in this study. C57BL/6NJ mice (RRID:IMSR_JAX:005304) were obtained at 6 weeks of age from The Jackson Laboratory. VIP/Peptide Histidine Isoleucine (PHI)+/+ (WT), heterozygous VIP/PHI± and knock-out VIP/PHI−/− (VIP-KO) mice were derived from breeding pairs of heterozygous VIP/PHI± mice provided by Dr. C. S. Colwell (University of California, Los Angeles). These mice had been raised on a C57BL/6 background (Colwell et al., 2003).

Animals were housed in translucent propylene cages (48 × 27 × 20 cm) and provided with *ad libitum* access to food and water. They were maintained in a 12/12 h light/dark cycle at a room temperature of 21°C. Mice were sacrificed at ZT14, at three to four months of age. This time was selected because previous studies indicated a substantial time of day effect of calcium binding proteins on the SCN connectome, with high fiber expression at ZT14 (LeSauter et al., 2009; Moore, 2016). Mice were deeply anesthetized with sodium pentobarbital (200 mg/kg) and perfused intracardially with saline followed by 4% paraformaldehyde in 0.1 M phosphate buffer, pH 7.3. All handling of animals was done in accordance with the Institutional Animal Care and Use Committee guidelines of the University.

### Antibodies

The primary antibodies were rabbit polyclonal anti-AVP (#20069, 1:5000, Immunostar, RRID:AB_572219); goat polyclonal anti-NP (sc-27093, Santa Cruz Biotechnology, RRID:AB_2061964); rabbit polyclonal anti-VIP (#20077, 1:5000, Immunostar, RRID:AB_572270), rabbit polyclonal anti-GRP (#20073, 1:2000, Immunostar, RRID:AB_572221), or rabbit polyclonal anti-ENK (#20065, 1:7500, Immunostar, RRID:AB_572250); goat polyclonal anti-CALR (#AB1550, 1:2000, Millipore Bioscience Research Reagents, RRID:AB_90764). Guinea pig polyclonal anti-AVP and anti-VIP (T-5048, RRID:AB_518680; and T-5030, RRID:AB_518690, Peninsula Laboratories) were also used in a few experimental runs with good results, similar to those with the rabbit antibodies and some sections were double-labeled with GRP-VIP using the anti-VIP made in guinea pig. New batches of the antibodies, however, gave very poor results and were not used further. In instances where all available primary antibodies were made in rabbit, we were not able to double-label the sections.

### Immunocytochemistry (ICC)

Brains were postfixed for 24 h at 4°C and then cryoprotected in 20% sucrose in 0.1 M phosphate buffer (PB) overnight. Coronal or sagittal sections (50 µm) were cut on a cryostat. Both single- and triple-label ICC was performed. We note that coronal sections are more familiar to students of the SCN than are sagittal sections. That said, in the coronal view, the connections along the rostral-caudal plane are severed. Because there is evidence that indicates the importance of the network along the rostro-caudal axis (Hazlerigg et al., 2005; Yan and Silver, 2008; Sosniyenko et al., 2009; Buijink et al., 2016), we used sagittal sections to investigate the network in this plane. We use coronal sections as well to enable reviewers to relate familiar coronal views of the nucleus to the less familiar sagittal view.

For single label immunofluorescence of brain sections were washed in 0.1 M PB with 0.1% Triton X-100 (0.1% PBT; Sigma-Aldrich), incubated in normal donkey serum (catalog #017-000-121 RRID:AB_2337258; Jackson ImmunoResearch) in 0.3% PBT for 1 h, and then incubated in rabbit anti-AVP at 4°C for 48 h. The sections were then washed in 0.1%PBT incubated in donkey anti rabbit secondary antibody conjugated to Cy2 fluorescent chromogen (RRID:AB_2340612, Jackson ImmunoResearch). Finally, sections were washed with 0.1 M PB, mounted, dehydrated in a series of 50–100% ethanol, and coverslipped with Krystalon (EMD Chemicals) and cover glass No. 1 (Fisher Scientific; catalog #12-544-18).

For triple-label immunofluorescence, sections were incubated in three primary antibodies raised in different species. Secondary antibodies were a mixture of Cy2 donkey anti-guinea pig (RRID:AB_2340467), Cy3 donkey anti-rabbit (RRID:AB_2340606), and Cy5 donkey anti-goat (RRID:AB_2340415) fluorescent chromogens (Jackson ImmunoResearch). Sections were washed, dehydrated, and coverslipped as above.

### Comparison of AVP staining in VIP-KO and WT

AVP cell counts in SCN, supraoptic nucleus (SON), and paraventricular nucleus (PVN), in WT and VIP-KO littermates, were studied in simultaneous immunostaining runs, using 50-µm coronal sections. For AVP cell counts in SCN of WT and colchicine-treated VIP-KO littermates, 50-µm sagittal sections were used. Photomicrographs of these areas were captured with a Nikon Eclipse E800 microscope (Nikon) equipped with a cooled CCD camera (Retiga Exi; Q-Imaging), using Q-capture software (RRID:SCR_014432, Q-Imaging) with the excitation wavelengths 480 ± 20 nm for Cy2. Images were stored as TIFF files with a 1392 × 1040-pixel array and then imported into Adobe Photoshop CS6 (Adobe Systems, Inc., RRID:SCR_014199). Counts were done independently by two researchers blind to the experimental conditions on three sections for each region with six mice/group and are reported as cell number/brain section. Inter-observer reliability was ≥93%.

AVP cell counts in WT and VIP-KO littermates were studied in a series of confocal images using ImageJ (National Institutes of Health; RRID:SCR_003070). The perimeter was measured on 1-µm optical sections in the largest extent of the neuron where a distinct nucleus was seen. The area through this plane was calculated from the perimeter.

### Appositions and colocalization

#### Confocal microscopy

Each triple-labeled section containing the SCN was observed under a Zeiss Axiovert 200 MOT fluorescence microscope with a Zeiss LSM 510 laser scanning confocal attachment (Carl Zeiss). The sections were excited with argon-krypton, argon, and helium-neon lasers using the excitation wavelengths of 488 nm for Cy2, 543 nm for Cy3, and 633 nm for Cy5. Each laser was excited sequentially to avoid cross talk between the three wavelengths.

#### Determination of appositions

**Figure 1.**
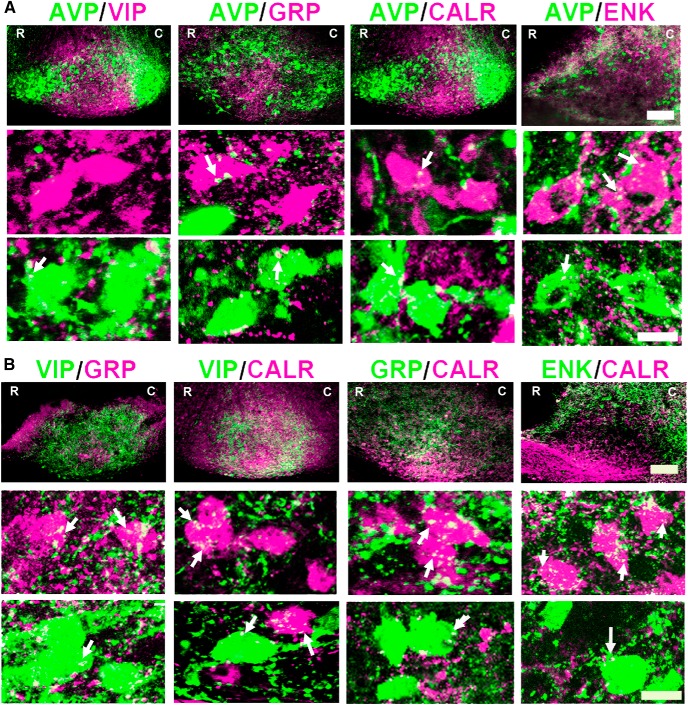
Photomicrographs of neuronal connections in the SCN of WT mice. ***A***, The images show the localization of neurons and fibers revealing AVP contacts onto VIP, GRP, CALR, and ENK neurons. ***B***, The images show the relationship between VIP and GRP, CALR and CALR with GRP and ENK. In both panels, the first row shows confocal images of the SCN as a whole to visualize the location of each of the cell types (*z*-axis: 8 µm; scale bar = 100 µm; R, rostral SCN; C, caudal SCN). The images in the second and third rows have been overexposed to optimize visualization of appositions of fibers onto neurons (arrows) for each of the peptides in the first row (*z*-axis = 2 µm; scale bars = 10 µm).

For visualization of the entire SCN, images were collected with a 20× objective as 8-µm multitract optical sections. For analysis of contacts among various peptidergic cell types, images were collected with a 63× objective (in Fig. 1, but not for the data analysis, images were overexposed to enhance visualization of the appositions). SCN neurons were examined through their entirety in 1-µm increments (*z*-axis), using the LSM 3.95 software (Carl Zeiss). Each neuronal cell body was examined to evaluate whether it bore zero, one to two, or more than or equal to three or more appositions, as in prior work (Jacomy et al., 1999; LeSauter et al., 2009). In addition, the total number of appositions made by AVP onto GRP, CALR, and ENK was assessed. There are caveats to the present methods of evaluating contacts, including (1) dendrites and axons cannot be differentiated, (2) synapses cannot be evaluated and (3) over or underestimation of the number of appositions due to ICC procedure, or (4) to missing fibers cut during sectioning, may occur as previously acknowledged (LeSauter et al., 2009). These do not affect the comparisons germane to the present study.

#### AVP in VIP-KO mice

The number of AVP neurons were compared in VIP-KO colchicine injected, and WT littermates (*N* = 6/group). VIP-KO mice were anesthetized, by intraperitoneal injection of 60 mg/kg ketamine and 5 mg/kg xylazine, placed into the stereotaxic apparatus (David Kopf Instruments), and prepared for aseptic surgery. A 10-µl Hamilton syringe (Hamilton) was used to inject 2 µl of colchicine (10 µg/µl in 0.9% saline; Sigma) in the lateral ventricle of VIP-KO animals and they were perfused 72 h later. Stereotaxic coordinates relative to bregma were: flat skull, anteroposterior, +1 mm; mediolateral, +0.7 mm; dorsoventral, -3.0 mm from the top of the skull. Sagittal sections from VIP-KO and WT controls were stained for AVP and cell counts were done as above with 90.3% interobserver agreement.

#### Determination of colocalization

To determine the colocalization of two peptides, neurons were examined in confocal scans and were considered double-labeled when coexpression was seen in at least three consecutive 1-µm scans.

### Statistical analysis

One-way ANOVA followed by Tukey’s *post hoc* test was used to compare the number of appositions made by each peptidergic cell type onto other peptidergic cell types (Fig. 4). Two-way ANOVA followed by Tukey’s *post hoc* test was used to compare the number of AVP cells in SCN, SON, and PVN of WT and VIP-KO littermate mice (Fig. 6), and to compare the number of AVP appositions onto other cell types in WT and VIP-KO mice (Fig. 7; Table 4); *t* tests were used for all other comparisons. All analyses were done using SigmaStat 2.03 (RRID:SCR_010285, SPSS Inc.).

## Results

### Appositions between peptidergic cell types in the SCN of WT mice

Contacts between peptidergic neuronal cell types were examined in WT mice, with the goal of determining whether appositions were reciprocal or not between specific cell types. The appositions made by each peptidergic neuron type onto the others were scored, where suitable antibodies were available. Low-power images of the entire SCN show the distribution of each peptide in neurons and fibers in sagittal sections of the SCN (Fig. 1*A*,*B*, top rows). The results indicate that AVP neurons are distributed throughout the shell and AVP fibers extend throughout the shell and the dorsal core. VIP neurons are located in the ventral core while GRP is mostly dorsal to the VIP cells. Both VIP and GRP fibers project throughout the SCN. CALR neurons lie primarily in the ventral SCN and fibers project dorsally throughout the nucleus. ENK neurons are sparsely distributed in the dorsal SCN and the fibers lie throughout the SCN with greatest density in the dorsal region. Figure 1, rows 2, shows examples of appositions of each immunoreactive fiber type studied onto AVP neurons, while Figure 1, rows 3, shows the reciprocal connections.

**Figure 2.**
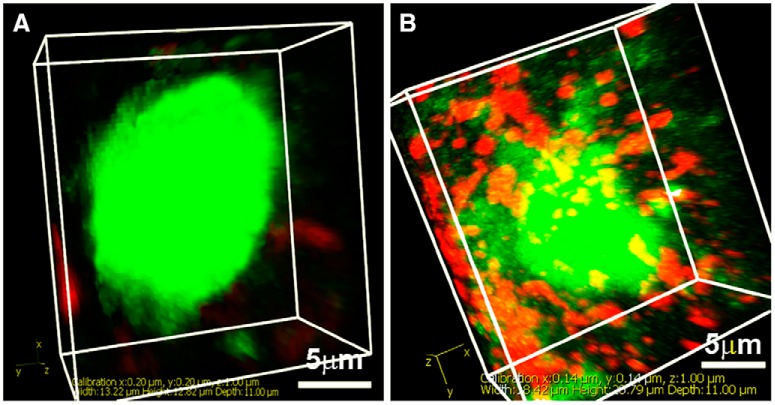
3D projection of images, each made from confocal stacks of 11 images of serial 1-µm slices, shows the range of appositions observed. ***A***, Image shows an example of an AVP neuron (green) lacking appositions from CALR neurons (red). ***B***, Image shows an AVP neuron (green) bearing numerous VIP (red) appositions.

**Figure. 3.**
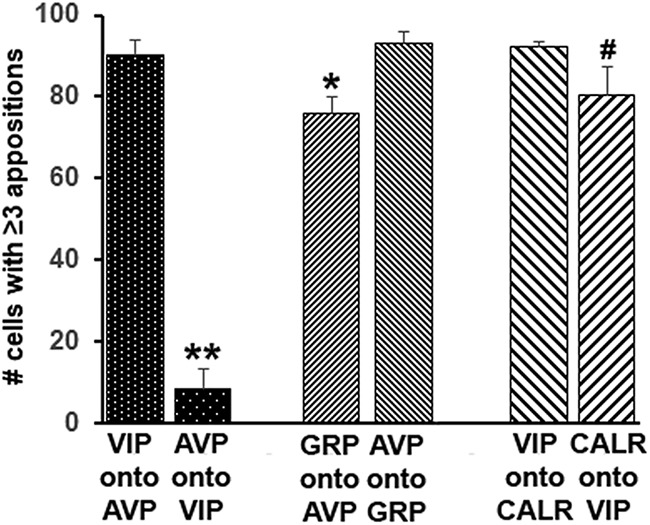
Number of neurons receiving more than or equal to three appositions. Here, instances of statistically significant differences between contacts made and received are shown, while Table 1 indicates statistical analysis for all appositions examined; **p < 0.001, *p = 0.02; #*p* = 0.06.

To quantify the appositions, contacts made by each neuronal type were evaluated using the following categories ≥3, 1–2, 0 as previously (Jacomy et al., 1999; LeSauter et al., 2002, 2009). Examples of zero and more than or equal to three appositions are shown in Figure 2. The results indicate that all neuronal cell types except one made numerous and reciprocal appositions onto each other. The most impressive exception was the paucity of connections from AVP fibers onto VIP neurons. In contrast, VIP neurons made numerous contacts onto AVP neurons. There were also significantly more appositions from AVP to GRP than conversely and a marginally greater number of appositions between VIP to CALR than conversely (Fig. 3).

### Appositions between peptidergic cell types in the SCN of VIP-KO mice

Because previous reports indicate that VIP mRNA is reduced at all times of day in VPAC2 mice (Harmar et al., 2002), we sought to determine the state of connections between AVP and other peptidergic cell types in our mice. To our surprise, there were no significant differences in reciprocal connections, detected between WT and VIP-KO mice. In the VIP-KO mouse, AVP to GRP appositions were somewhat more numerous than GRP to AVP, but unlike the WT, this difference was not significant.

**Figure 4.**
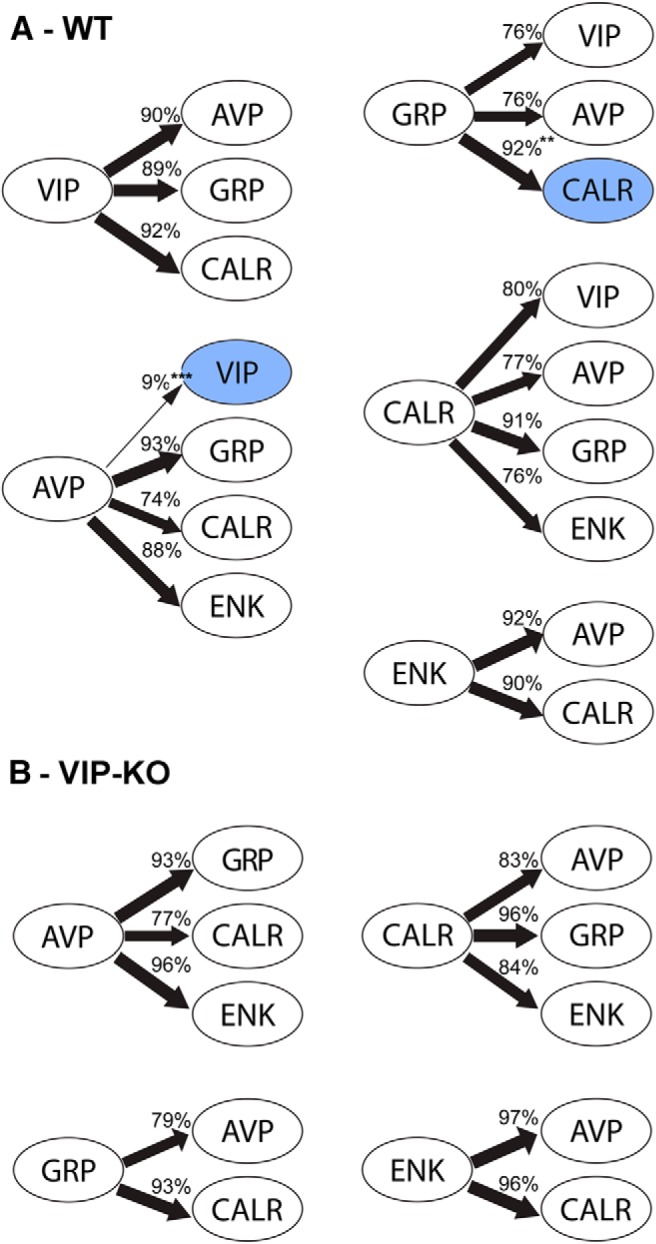
Schematic representations of the connections between peptidergic neuronal types. The numbers and the arrow thickness indicate the proportion of cells receiving more than or equal to three appositions (statistics in Tables 2, 3). ***A***, In WT mice, there was a preferential unilateral direction of communication between AVP-VIP and GRP-CALR (blue). While the other connections indicated are all reciprocal, there are few AVP to VIP contacts and more GRP to CALR contacts than the converse (***p < 0.001, **p = 0.008). ***B***, In contrast to WT mice, in VIP-KO mice, there was no evidence of preferential direction of communication between peptidergic cell types.

We next asked whether a particular peptidergic SCN neuron made selective contacts or communicated equally with all other types [Fig. 4*A*,*B*; Tables 1 (WT), 2 (VIP-KO)]. This assessment of appositions shows that AVP fibers contact fewer VIP cells than GRP, CALR, or ENK cells in the WT mouse (*F*_(3,29)_ = 20.2, *p* < 0.001). Also, there is a significantly greater number of appositions of GRP onto CALR neurons (*F*_(2,23)_ = 6.2, *p* = 0.008) compared to AVP neurons, although this statistical difference did not hold for GRP onto VIP. In contrast, VIP, ENK, and CALR cells make a similar number of appositions with the other cell types.

**Table 1.** Percentage of neurons receiving appositions from other neuronal cell types per WT mouse (mean ± SEM)

Cell type	% cells with ≥3 appositions	% cells with 1–2 appositions	% cells with 0 appositions	# cells	# animals	*t* test	*p* value
AVP to VIP	8.5 ± 4.9	19.5 ± 7.7	72.0 ± 10.8	251	6		
VIP to AVP	90.3 ± 3.8	8.9 ± 3.6	0.8 ± 0.3	458	5	*t*_(9)_ = 11.5	<0.001
AVP to GRP	93.1 ± 2.7	6.9 ± 2.7	0	88	5		
GRP to AVP	75.8 ± 4.0	17.9 ± 3.7	6.3 ± 1.8	446	11	*t*_(14)_ = 2.63	0.02
AVP to CALR	73.6 ± 8.2	7.8 ± 2.3	18.7 ± 7.3	1044	12		
CALR to AVP	77.2 ± 5.4	6.5 ± 1.	15.9 ± 5.1	813	11	*t*_(21)_ = 0.38	0.71
AVP to ENK	87.8 ± 4.7	6.6 ± 2.4	5.6 ± 3.5	121	7		
ENK to AVP	91.5 ± 2.8	7.2 ± 2.3	1.3 ± 0.8	381	7	*t*_(12)_ = 0.63	0.54
VIP to GRP	88.9 ± 2.4	7.7 ± 1.4	3.5 ± 1.6	61	3		
GRP to VIP	75.8 ± 6.6	11.6 ± 1.7	12.6 ± 4.9	148	3	*t*_(4)_ = 1.52	0.20
VIP to CALR	92.4 ± 0.9	5.2 ± 0.4	2.3 ± 0.8	988	8		
CALR to VIP	80.3 ± 7.2	13.5 ± 8.2	6.2 ± 1.2	229	4	*t*_(10)_ = 2.11	0.06
GRP to CALR	92.3 ± 2.4	4.0 ± 1.3	3.7 ± 1.3	953	10		
CALR to GRP	90.5 ± 5.4	7.0 ± 4.6	2.5 ± 1.6	99	6	*t*_(14)_ = 0.43	0.67
ENK to CALR	89.7 ± 5.5	7.8 ± 4.3	2.4 ± 1.5	432	6		
CALR to ENK	76.2 ± 15.4	9.8 ± 5.4	14.1 ± 1.1	98	5	*t*_(9)_ = 0.8	0.44

Statistical analysis tests for preferential direction of communications between neuronal cell types for more than or equal to three appositions.

Unlike the WT mouse, there were no significant differences in reciprocal connections between GRP and AVP and CALR cell types in the VIP-KO mouse. Comparison of WT and VIP-KO in number of neurons receiving appositions from other neuronal cell types yielded no significant differences between strains (Table 3).

**Table 2.** Average percentage of neurons receiving appositions from other neuronal cell types per VIP-KO mouse (mean ± SEM)

Cell type	% cells with ≥ 3 appositions	% cells with 1–2 appositions	% cells with 0 appositions	# cells	# animals	*t* test	*p* value
AVP to GRP	93.0 ± 5.0	7.0 ± 5.0	0	76	5		
GRP to AVP	79.0 ± 10.7	6.5 ± 5.4	14.6 ± 7.4	152	4	*t*_(7)_ = 1.1	0.23
AVP to CALR	77.0 ± 9.6	4.3 ± 2.1	18.7 ± 7.7	720	8		
CALR to AVP	82.6 ± 3.7	5.8 ± 1.8	11.6 ± 3.9	549	8	*t*_(14)_ = 0.5	0.62
AVP to ENK	96.0 ± 1.9	3.3 ± 1.6	1.2 ± 0.5	218	5		
ENK to AVP	97.1 ± 1.7	2.9 ± 1.7	0	222	5	*t*_(8)_ = 0.6	0.59
GRP to CALR	93.0 ± 2.5	4.0 ± 1.9	3.0 ± 1.8	684	8	*t*_(14)_ = 0.6 *	0.58
CALR to GRP	95.8 ± 3.9	0	4.2 ± 3.9	109	8	*T*_(8,8)_ = 51	0.08
ENK to CALR	96.2 ± 1.7	2.7 ± 1.1	1.1 ± 0.7	510	4	*t*_(6)_ = 1.4*	0.23
CLR to ENK	83.6 ± 7.9	13.6 ± 6.3	2.9 ± 1.8	133	4	*T*_(4,4)_ = 20	0.69

Statistical analysis tests for preferential direction of communications between neuronal cell types for more than or equal to three appositions. * Normality or equal variance failed, Mann–Whitney rank test (*T*) is shown.

**Table 3.** Difference between WT and VIP-KO in % neurons receiving more than or equal to three appositions

Cell type	*t* test WT vs VIP-KO	*p* value
AVP to GRP	*t*_(8)_ = 0.02	0.98
GRP to AVP	*t*_(13)_ = 0.32	0.75
AVP to CALR	*t*_(18)_ = 0.26	0.80
CALR to AVP	*t*_(17)_ = 0.68	0.50
AVP to ENK	*t*_(10)_ = 1.21	0.26
ENK to AVP	*t*_(10)_ = 1.40	0.19
GRP to CALR	*t*_(16)_ = 0.02	0.98
CALR to GRP	*t*_(12)_ = 0.42	0.68
ENK to CALR	*t*_(8)_ = 0.84	0.43
CALR to ENK	*t*_(7)_ = 1.02	0.34

Data for each group is shown in Tables 1 (WT) and 2 (VIP-KO).

### Effect of VIP-KO on AVP

**Figure 5.**
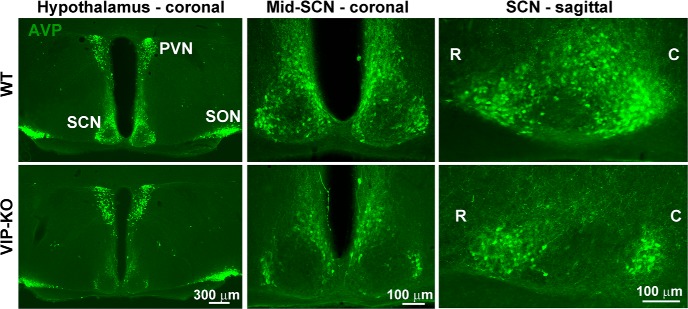
Photomicrographs depict AVP-ir in coronal and sagittal views of the SCN in littermates of WT (top row) and VIP-KO (bottom row) mice tested in the same experimental runs. While AVP expression is similar in PVN and SON of WT and VIP-KO mice, it is reduced in SCN of the mutants (R, rostral; C, caudal).

**Figure 6.**
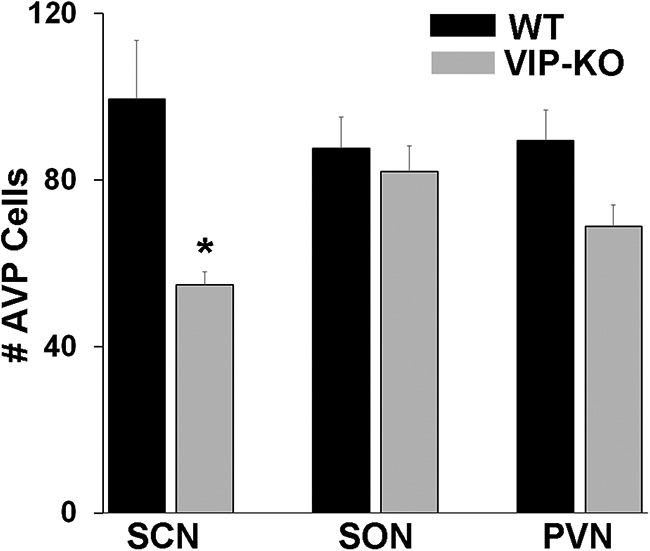
Numbers of AVP neurons in SCN, SON and PVN of WT and VIP-KO mice. In VIP-KO, the number of AVP-ir neurons is significantly lower in the SCN, but not in SON or PVN compared to WT littermates; *p < 0.001 versus WT.

When examining appositions, we noted that, compared to WT mice, AVP expression was reduced in the SCN neurons of VIP-KO mice, with no differences between groups in the size of SCN AVP cells (WT: 84.2 ± 1.6 µm^2^; VIP-KO: 86.7 ± 2.3 µm^2^, *t*_(133)_ = 0.88, *p* = 0.38). Therefore, AVP expression in littermates of the WT and VIP-KO mouse were compared. Examination of coronal and sagittal sections indicates that the expression of AVP in the VIP-KO mouse is severely reduced in the SCN. This can be seen throughout the extent of the nucleus in photomicrographs of sections stained for AVP (Fig. 5). Comparison of the number of AVP neurons in SCN, SON and PVN indicates that the reduction in AVP-ir cell number is restricted to the SCN and is not seen in SON and PVN, nearby AVP-rich regions (ANOVA: WT vs VIP-KO: *F*_(1,33)_ = 11.3, *p* = 0.002; brain regions *F*_(2,33)_ = 0.46, *p* = 0.63; interaction *F*_(2,33)_ = 2.8, *p* = 0,08; Fig. 6).

### AVP appositions onto other cell types, WT versus VIP-KO

**Figure 7.**
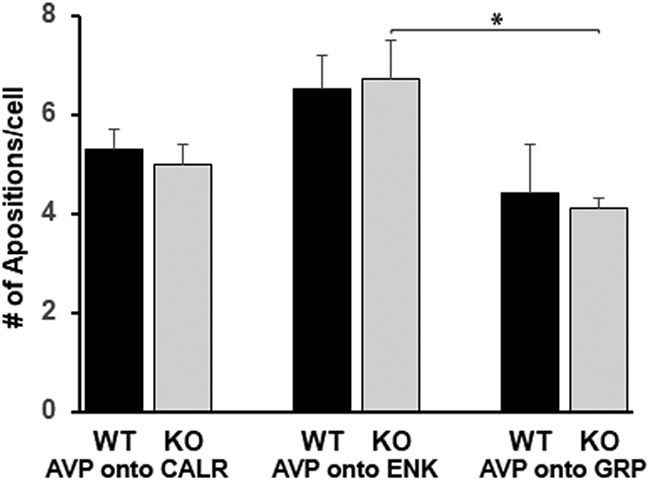
Quantification of number of appositions of AVP cells onto other peptidergic cell types in WT and VIP-KO mice (ANOVA: WT vs VIP-KO: *F*_(1,30)_ = 0.07, *p* = 0.79; peptidergic cell type *F*_(2,30)_ = 7.94, *p* = 0.002; interaction *F*_(2,30)_ = 0.45, *p* = 0.89). * Tukey test: *p* = 0.01.

We were surprised to note that although VIP-KO mice had fewer AVP-ir neurons, the number of GRP, CALR and ENK neurons receiving more than or equal to three AVP appositions did not differ between WT and VIP-KO mice. To reassess this surprising result, we revisited the finding by analyzing the absolute number of appositions made by AVP cells onto ENK, GRP, and CALR cells in WT and VIP-KO littermates (Fig. 7; Table 4). Here again, there were no differences between WT and VIP-KO mice in number of AVP appositions made on each peptidergic type.

**Table 4.** Comparison of WT and VIP-KO mice: number of AVP appositions/cell onto CALR, ENK, and GRP cell types

Cell type	# appositions/cell (mean ± SEM)	# cells	# animals	Tukey test WT vs VIP-KO
AVP to CALR-WT	5.3 ± 0.4	222	6	*p* = 0.73
AVP to CALR-KO	5.0 ± 0.4	189	6
AVP to ENK-WT	6.5 ± 0.7	121	5	*p* = 0.80
AVP to ENK-KO	6.7 ± 0.8	178	5
AVP to GRP-WT	4.4 ± 1.0	70	4	*p* = 0.70
AVP to GRP-KO	4.1 ± 0.2	68	5	

### Number of AVP neurons in SCN of colchicine-treated VIP-KO mice

**Figure 8.**
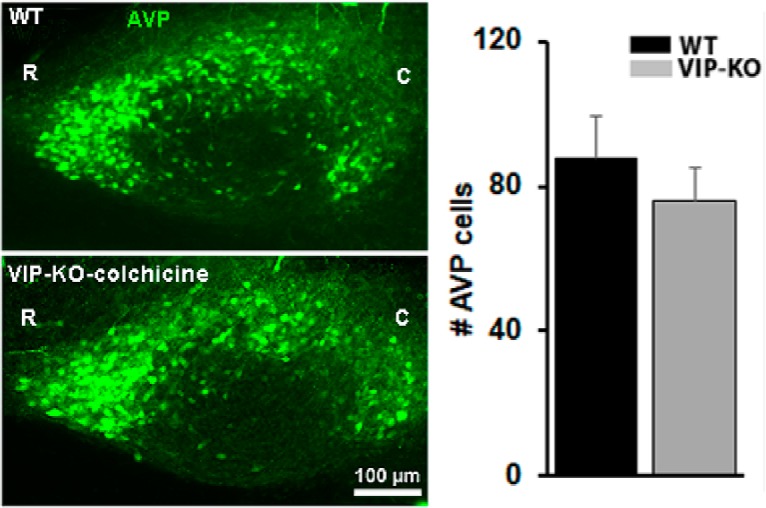
Left panels: Photomicrographs depict AVP in SCN of WT and VIP-KO colchicine-treated littermates (R, rostral; C, caudal). Each pair of animals (WT and VIP-KO-colchicine) was tested in the same experimental runs. Right panels: Number of AVP cells in WT and VIP-KO colchicine treated mice (*t*_(10)_ = 0.82, *p* = 0.43).

The assessment of appositions indicated similar numbers of contacts between AVP and other neurons in the VIP-KO and WT animals, although we saw fewer AVP neurons in the VIP-KO. We sought to assess whether this was the result of fewer AVP neurons making more contacts, or a similar number of neurons in the two cell types but a reduction in detectable AVP peptide. To assess whether there is a decrease in numbers of neurons producing AVP in the mutant mice or rather, a deficit in AVP synthesis, the number of AVP neurons in colchicine-treated VIP-KO mice and WT littermates were compared (*N* = 6/group; Fig. 8). The results indicate that there was no significant difference in the number of AVP neurons in the SCN of WT and colchicine-treated VIP-KO littermate mice.

### Colocalization of peptides in WT and VIP-KO

**Figure 9.**
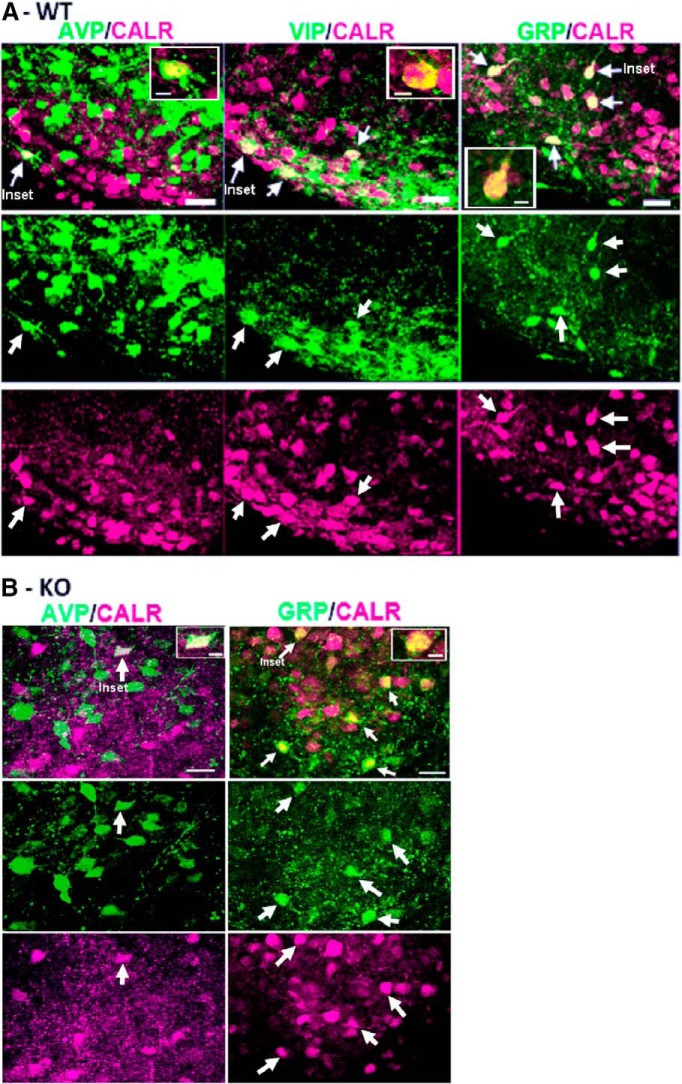
Confocal photomicrographs of sagittal SCN sections from WT (***A***) or VIP-KO (***B***) mouse stained for AVP, CALR, VIP, and GRP, as indicated. The merged channels are shown in the upper rows and each separate channel is shown in the middle and third rows (*z*-axis = 3 µm, scale bar = 20 µm). The arrows point to double-labeled cells, In each panel in the merged channel, the cell shown in the inset is also identified (insets: *z*-axis = 1 µm; scale bar = 5 µm).

**Figure 10.**
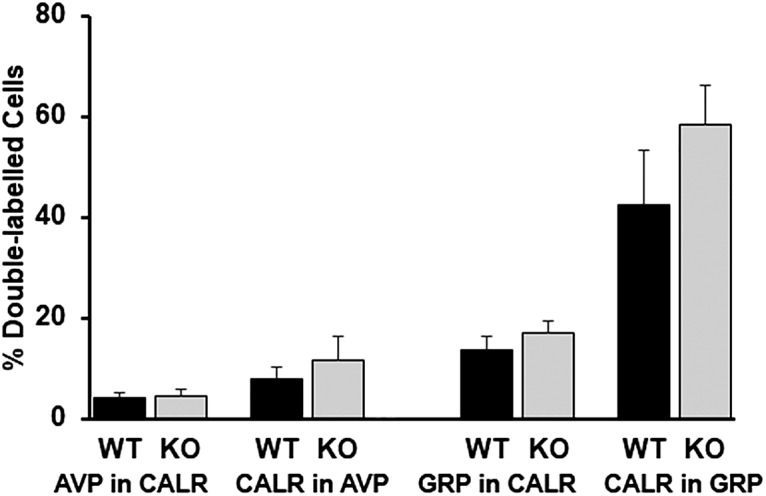
Colocalization of peptides in WT and VIP-KO mice. No differences were detected between genotypes. See Table 5 for statistics.

We next asked whether VIP-KO and WT mice differed in colocalization of major SCN peptides. In WT mice, there was colocalization of CALR with AVP, VIP, and GRP (Fig. 9). There was no colocalization detected of other peptides (AVP/GRP, AVP/VIP, VIP/GRP AND ENK/AVP, ENK/GRP, ENK/VIP, or ENK/CALR; data not shown). Finally, there were no differences between WT and VIP-KO littermates in colocalization of peptides (Fig. 10; Table 5).

**Table 5.** Comparison of WT and VIP-KO: % neurons colocalizing AVP, CALR, GRP

Colocalization	WT (% cells ± SEM)	# cells	# animals	KO (% cells ± SEM)	# cells	# animals	*t* test	*p* value
AVP in CALR	4.1 ± 1.0	1060	12	4.4 ± 1.3	750	8	*t*_(18)_ = 0.21	0.83
CALR in AVP	8.0 ± 2.3	879	12	11.6 ± 4.7	579	8	*t*_(18)_ = 0.75* T_(8,12)_ = 88	0.46 0.79
GRP in CALR	13.6 ± 2.9	1123	10	16.9 ± 2.4	831	8	*t*_(16)_ = 0.83	0.42
CALR in GRP	42.6 ± 10.7	290	10	58.3 ± 7.9	256	8	*t*_(16)_ = 1.12	0.28
VIP in CALR	6.9 ± 1.8	988	8					
CALR in VIP	15 ± 1.8	307	8					

* Normality failed, Mann–Whitney rank test (*T*) is shown.

## Discussion

### Appositions between SCN neurons

Although individual neurons are oscillators, it is widely agreed that the network of the SCN is essential for normal circadian timekeeping (Hastings et al., 2014; Pauls et al., 2016; Herzog et al., 2017). This study addressed a caveat in that much of the data on which this consensus rests derives from studies of mice, but evidence on the nature of connections among SCN neurons derives from rats and/or hamsters. The general notion is that in rodents, core neurons communicate with those in the shell while there is less communication in the reverse direction (Leak et al., 1999). The present study explores appositions between various core and shell peptidergic cell types in the mouse SCN. The results suggest that while approximately equal, reciprocal appositions occur between some neurons examined, impressively, this was not the case for other, major core-shell connections. Specifically, for AVP to VIP, fewer than 10% of VIP neurons received more than or equal to three AVP contacts, while for VIP to AVP, 90% of AVP neurons received more than or equal to three VIP contacts. Some differential communication among core neurons was also detected, with GRP making more appositions onto CalR than onto VIP or AVP neurons.

We also found that in contrast to their WT littermates, there was a marked reduction in number of detectable AVP neurons in VIP-KO mice, although a few intensely labeled neurons were seen in each animal. Surprisingly, these mice had the same numbers of appositions as WT mice. We assessed whether this could be due to sprouting of fibers as a consequence of the reduction in neuron number in these mice. However, when transport of AVP was blocked by administration of colchicine in VIP-KO mice, the numbers of AVP neurons were comparable to WT animals. Such results are consistent with reduction of AVP synthesis and/or asynchronous AVP rhythms in the VIP-KO mice and with findings that VIP regulates the long-term firing rate of SCN neurons (Kudo et al., 2013)

### The SCN connectome

The results of this study clarify core to shell communication in the mouse SCN and reexamine the general hypothesis put forth in previous work that the communication from core to shell is more extensive than the reverse (Daikoku et al., 1992; Leak et al., 1999). The results indicate that this general pattern does not apply to all neuronal subtypes of the core. More specifically, we find that there are far more contacts made by VIP core neurons onto AVP shell neurons than the converse. There was also evidence of specialization in that the core GRP neurons receive more appositions from AVP neurons than the converse. Unexpected was the trend suggesting that essentially all CalR neurons receive appositions from GRP, but there are fewer contacts in the reverse direction. Such results suggest important specializations of the network. The same general pattern of core to shell and intracore connections is seen in other species, with differences in the specific peptides involved. In hamster, CalB neurons receive numerous appositions from VIP and GRP fibers (LeSauter et al., 2002). Reciprocal connections are seen between VIP and GRP neurons in hamsters and in rats (Romijn et al., 1997). It is clear from numerous studies that for AVP-to-VIP connections, AVP fibers are much more densely distributed in the AVP-rich shell area than in the VIP and GRP-rich core areas both in rats and in mice (van den Pol, 1986; Daikoku et al., 1992; Abrahamson and Moore, 2001; Moore et al., 2002). In close agreement with the present results, Jacomy et al. (1999) show that around 80% of VIP cells received few AVP appositions. With regard to the present results, we note that this work does not specify whether all appositions reported here originate from cells local to the SCN as cross talk between the bilateral SCN (Michel et al., 2013) or input from other brain regions may also occur.

Temporal variation of peptide expression complicates the task of defining the SCN connectome. Expression levels of some SCN peptides are under circadian regulation, with possible species differences in times of peak expression among peptides. Circadian trafficking of calcium binding proteins in fibers of SCN neurons has been demonstrated in hamster (LeSauter et al., 2009) and rat (Moore, 2016). AVP receptor expression and AVP, VIP and GRP content in the SCN are rhythmic (Okamoto et al., 1991; Inouye and Shibata, 1994; Kalsbeek et al., 2010).

### VIP regulation of AVP

Our observation that AVP protein is reduced in VIP-KO mice is consistent with previous related reports. VPAC2 is found in nearly all SCN cells, including AVP-containing cells (An et al., 2012). AVP mRNA is reduced in VPAC2-KO mice (Harmar et al., 2002) and AVP is induced by VIP or VPAC2 agonist (Rusnak et al., 2007). We had been surprised to note that the number of AVP appositions on other neurons did not differ between VIP-KO and WT mice. Given the finding that in colchicine-treated VIP-KO mice the number of AVP neurons was similar to their WT littermates, it appears that the VIP-KO mice have a reduction in AVP protein synthesis, but the amount of the peptide produced is sufficient to be detected with the present protocols.

### VIP-KO alters other genes and proteins

It appears that the altered capability of VIP-KO and VPAC2-KO mice ability to express rhythmic behavioral responses is due in part to disruption of normal signaling not only of VIP but also of AVP. AVP not only augments the amplitude of rhythms within SCN but also acts as an output signal to the rest of the brain. There is a circadian rhythm of AVP in the cerebrospinal fluid (Schwartz and Reppert, 1985). AVP, acting through its V1 receptor, is important in augmenting electrical activity of SCN neurons (Ingram et al., 1998). AVP participates in coordinating oscillations through the AVP V1a receptor which extend widely in the SCN, including both core and shell regions (Li et al., 2009). AVP input to SCN neurons may contribute to its synchronizing effect within the SCN (Ingram et al., 1998; Bittman, 2009; Li et al., 2009), and this is likely part of the mechanism whereby AVP influences locomotor rhythms (Cormier et al., 2015). In summary, we conclude that the well characterized arrhythmicity of mice lacking VIP or its receptor (Colwell et al., 2003; Brown et al., 2007; Hughes and Piggins, 2008) may be due in part to the disruption in AVP in these animals.

### Colocalization of peptides in the SCN is the rule and not an exception

The present results indicate that VIP and CALR as well as GRP and CALR are coexpressed in some but not all SCN neurons. In rat, colocalization of VIP, PHI, and GRP in some but not all neurons have been reported (Okamura and Ibata, 1986; Kawamoto et al., 2003). In hamster analysis of colocalization of peptides shows that 91% of the substance P cells, 42% of the GRP cells and 60% of the VIP cells in the core coexpress CalB (LeSauter et al., 2002).

### The SCN is a dynamic network

The work on appositions in the WT mouse indicates that the SCN network is highly specialized. While AVP contacts onto VIP neurons are very sparse, the numbers of contacts onto GRP neurons are somewhat augmented compared to communications in the reverse direction. For other neuronal types, the communications appear to be largely reciprocal. The connectome delineated here indicates that the peptidergic network in VIP-KO animals is similar to that of WT, although the VIP protein is absent. Furthermore, in VIP-KO animals, there is a deficit not only in the VIP protein, but also in AVP. The consequences of compromised VIP-ergic signaling in the SCN have been seen in altered behavioral, cellular, and intercellular circadian activity. The present study shows that AVP synthesis is also compromised in these KO mice. Taken together this study characterizes the SCN network in mouse and further highlights the interrelationship between VIP and AVP in maintaining circadian timekeeping.

## Synthesis

Reviewing Editor: Douglas Bayliss, University of Virginia School of Medicine Decisions are customarily a result of the Reviewing Editor and the peer reviewers coming together and discussing their recommendations until a consensus is reached. When revisions are invited, a fact-based synthesis statement explaining their decision and outlining what is needed to prepare a revision will be listed below. The following reviewer(s) agreed to reveal their identity: Christopher Colwell.

This manuscript was seen by two reviewers, both of whom found that this analysis of biased projections between specific peptidergic cell types of the mouse SCN results was carefully performed and provides useful new information. As you will see from the comments listed below, they have offered are a number of suggestions that you should consider in your revision of this manuscript; these are largely minor, involving changes in presentation and clarifications of methods and interpretations. After consultation, the reviewers felt that a more appropriate comparison for colchicine-treated VIP KO would be colchicine-treated WT (as opposed to, or in addition to, untreated WT); your response should deal with this issue, preferably by experiment.

Reviewer #1

Advances the Field

Minor advances, adding to previously reported data.

Statistics

If the data was normally distributed and had equal variance, the parametric tests used are ok (addressed in the comments to the authors)

Visual Abstract

The visual abstract is a nice illustration of the topic and WT results, but the question marks should be replaced with KO results.

Comments to the Authors

The authors analyzed projections between specific peptidergic cell types of the SCN and compared them to the KO for one of these cell groups. While I like the topic and the results, I think the presentation of the data can be improved, and the data could be expanded to include those connections that were not addressed.

General comments:

- Please use “cell type” when referring to cell populations instead of “phenotype”.

- While appositions are an indication of actual synaptic contacts, they could also be in close proximity, but non-synaptic. Therefore, referring to these appositions as “connections” is not perfectly precise. What is shown here is also not exactly the “connectome”, since that would require all connections of the SCN (including ENK-GRP, ENK-VIP, and more). The title and text should therefore avoid the word “connectome”.

- Could you also analyze the missing ENK-GRP and ENK-VIP connections?

- Why were the other cell types mentioned in the introduction (SS, GAD, PHI, NPY, 5HT, CCK) not part of the analysis?

Introduction:

- Line 89: more precisely among different SCN cell types

- Line 99, 107: keep GRP abbreviated

- Line 139: use only one of the words, determine or assess

Methods:

- Why were some sections sagittal, and others coronal?

- Shouldn't appositions onto dendrites be important as well, in addition to those on the soma?

- Did the few runs with good results using guinea pig antibodies show similar results as those you report?

- Could you measure cell sizes as 3D volumes, not just section areas (since you have 3D stacks)? 3D reconstructions of appositions would also help a lot.

- You used parametric tests. Was your data normally distributed? Did they have equal variance?

- Line 187: washed; line 243: ventricle of

Results:

- Since colchicine restored AVP cell number, wouldn't another interpretation be that AVP is not synthesized less, but transported faster away from the soma to the presynapse in VIP-KO?

- Lines 305-310: This belongs to the VIP-KO section.

- Lines 366, 387: Please add your data (images showing no difference between genotypes, images showing no colocalization and no differences in colocalization).

Discussion:

- Line 455: Do Avp cell numbers recover in another circadian phase in the VIP-KO (without colchicine)?

- Line 528: What is similar?

Figures:

- The visual abstract should show the results of the VIP-KO experiments instead of question marks.

- Fig.1: Please add directional labels to the top rows. The signals in the 2nd and 3rd rows are overexposed, so please use a higher maximum level for your intensity ranges. Add arrows to where you identified appositions. 3D reconstructions of such datasets would help illustrate the spatial relations and morphologies of putative contact sites. Line 293: and for CALR with GRP and ENK.

- Fig.3: Please add what the blue label means. Line 329: Please describe in more detail where the preferred unilateral direction can be seen.

- Fig.4: Please add directional labels to the sagittal images. The minimum level in your intensity range should be adjusted to reduce the background. Please add a green label “AVP” to the figure.

- Fig.7: Please add directional labels. In the top image, cells seem accumulated on the right side, while they are accumulated on the left side in the bottom image. Are rostral and caudal reversed?

- Fig.8: Please show split channels as well. Line 393: remove the repeated “confocal scans”.

- You only show WT images, please also add images from your VIP-KO data.

- Please convert green-red images to green-magenta for color-blind readers and better color contrast.

- Table 3: There seems to be another column here that was taken out, but its values are still visible.

Reviewer #2

Advances the Field

Most of our modern understanding of the SCN circuit comes from use of genetically manipulated mice. Yet most of the anatomy of the SCN is based on work in rats and hamsters. The present study helps bridge this gap with a detailed analysis of the synaptic contacts made between various SCN peptidergic neuronal types in WT and VIO-KO mice..

Visual Abstract

I think that this is a clear summary of the author's model.

Comments to the Authors

Most of our modern understanding of the SCN circuit comes from use of genetically manipulated mice. Yet most of the anatomy of the SCN is based on work in rats and hamsters. The present study helps bridge this gap with a detailed analysis of the synaptic contacts made between various SCN peptidergic neuronal types in WT and VIO-KO mice. The authors report that the connectivity between AVP and VIP neurons are biased i.e. AVP neurons make significantly more contacts onto GRP neurons and significantly fewer onto VIP neurons than conversely. The other SCN peptidergic phenotypes make largely reciprocal appositions. Analysis of the SCN in VIP-KO mice indicates that there is a substantial reduction in AVP that is specific to the SCN. Overall the work delineates the connections among SCN neurons in the SCN network and demonstrates an unexpected complexity in interpreting the consequences of gene knockouts.

In generally, this is a very nice study with clear presentation of the findings and articulate writing. The experiments are well laid out and results completely believable. The graphic abstract was very helpful. The authors make a clear argument for the importance of publishing this information about the mouse model.

In Fig. 1, the photomicrographs appear to be over-exposed. Maybe this is necessary to see the appositions, and then the images can be left as they are now, however in some of the micrographs is hard to appreciate the overlaps. The authors should add arrows to this figure (similar to Fig 8) showing what they considered as appositions, this will help the readers. It would be helpful to see some examples of the cells with different numbers of appositions.

In Fig. 2, the authors only show quantification for '3 combinations', is there a reason for this choice?

The organization of the manuscript was sometimes confusing. To provide two examples:

1) Page 13: This paragraph or some version of it might be better in the results than in the methods, it very well describes the rationale behind the authors extensive data analyses. “The assessment of appositions indicated similar numbers of contacts between AVP and other neurons in the VIP-KO and WT animals, even though we saw fewer AVP neurons in the VIP-KO. We sought to assess whether this was the result of fewer AP neurons making more contacts, or a similar number of neurons in the two phenotypes but a reduction in detectable AVP peptide. To this end, AVP cell numbers were compared in VIP-KO colchicine injected, and WT littermates (N=6/group).”

2) Page 18, the paragraph after Fig 2, “Because the VIP-KO mice had fewer AVP-ir neurons in the SCN, we next sought to determine the state of connections between AVP and other peptidergic cell types in these mice (Table 2). There were no significant differences in reciprocal connections, detected between WT and VIP-KO mice. In the VIP-KO mouse, AVP to GRP appositions were somewhat more numerous than GRP to AVP, but unlike the WT, this difference was not significant.” does not seem to belong here, as this paragraph is describing the WT finding (as described in the title), and the same description is continued in the beginning of the paragraph below, maybe would be better in the paragraph below after the description of the data in fig2? The authors may want to first introduce the rationale behind using the VIP-KO model.

Fig 7: Shouldn't the colchicine treated VIP KO be compared to both colchicine-treated WT and untreated WT? Also, for those readers less familiar with neuroanatomical techniques, the authors should explain why colchicine was used in the methods or results.

Fig 8, it looks to us like the AVP panel is overexposed.

We had a few other minor issues.

“mice lacking VIP in the SCN have abnormal circadian activity, impaired cellular rhythmicity, and reduced synchrony among neurons (Aton et al., 2005; Brown et al., 2007).” Please add the Colwell et al., 2003 citation. This is the first report of the VIP KO line used in the present study but also contains more detailed behavioral analysis that was done in other studies.

In the discussion, the authors suggest that their results are consistent with asynchronous AVP rhythms in the VIP-KO mice but do not have much support for this. An alternative explanation is that the impact of VIP on excitability of AVP neurons (Kudo et al., 2013) may also contribute to the reduced expression. But since the mechanisms by which the loss of VIP would impact expression of AVP are unknown it may be better to just state this.

Kudo T, Tahara Y, Gamble KL, McMahon DG, Block GD, Colwell CS. Vasoactive intestinal peptide produces long-lasting changes in neural activity in the suprachiasmatic nucleus. J Neurophysiol. 2013 110(5):1097-106

In the discussion, the authors make the point that these peptides are rhythmically expressed but do not reflect on how this might impact the interpretations of their analysis. A sentence needs to be added to the effect that they cannot exclude the possibility that these relationships could vary with time. They controlled for this possible variation by keeping the time of tissue collection constant.

The statement that “we conclude that the well characterized arrhythmicity of mice lacking VIP or its receptor (Colwell et al., 2003; Brown et al., 2007; Hughes & Piggins, 2008) is due in part to the disruption in AVP in these animals” is too strong. We agree that their finding raise the possibility that the reduction of AVP contributes to the physiological and behavior phenotype. The authors could just add “may be due in part”. Also, what about the VPAC2 KO studies?

Can the authors please replace the word co-localization with co-expression or co-labelling, the latter are more appropriate when describing the images in results and Fig legends as based on the images shown, their magnification is not high enough to show the localization of the staining in the intracellular compartments, clearly there is overlap in some parts but that does not necessarily mean that the labelled proteins are in the same cell compartment. For instance: in the methods paragraph page 14, page 24, Table 5, Fig 9.

Please indicate location (anterior or posterior SCN) of the coronal section authors used in this study.

We could not find a citation to “Fig 2” in the result section.

The photographs of WT VIP sagittal section in the Fig. 4 and Fig.7 look the same. Did authors perform IHC at the same time in WT, VIP KO, and VIP KO with colchicine?

line 305, “Because the VIP-KO mice had fewer AVP-ir neurons in the SCN,” appears premature as the AVP data of VIP KO will come later.

In the overall depiction of the SCN connectome in the discussion, the authors may want to consider crosstalk between the SCN and other brain regions. The “high mag” focus on cellular appositions is useful for understanding SCN circuitry, but runs the risk of ignoring extra-SCN inputs as well as feedback. How can the authors be certain that all cellular contacts in question originate from SCN neurons given that these peptides are also expressed outside the SCN? The authors are not using genetic drivers to restrict fluorescent expression to SCN neurons, so regional specificity needs to be addressed.

The authors could consider previously characterized connections between left and right SCN (inter-SCN coupling) mediated by GABA, VIP, and AVP.

Michel, S. Marek, R. , vanderLeest, H. T., vanSteensel, M. J., Schwartz, W. J., Colwell, C. S. and Meijer, J. H. (2013), Mechanism of bilateral communication in the suprachiasmatic nucleus. Eur J Neurosci, 37: 964-971. doi:10.1111/ejn.12109.
